# Morphological Traits Evaluated with Random Forest Method Explains Natural Classification of Grapevine (*Vitis vinifera* L.) Cultivars

**DOI:** 10.3390/plants11243428

**Published:** 2022-12-08

**Authors:** Zsófia Szűgyi-Reiczigel, Márta Ladányi, György Dénes Bisztray, Zsuzsanna Varga, Péter Bodor-Pesti

**Affiliations:** 1 Department of Applied Statistics, Institute of Mathematics and Basic Science, University of Agriculture and Life Sciences, Villányi út 29-43, 1118 Budapest, Hungary; 2 Department of Viticulture, Institute for Viticulture and Oenology, Hungarian University of Agriculture and Life Sciences, Villányi út 29-43, 1118 Budapest, Hungary

**Keywords:** *Vitis vinifera* L., ampelography, numerical morphology, variable selection, random forest

## Abstract

There are hundreds of morphologic and morphometric traits available to classify and identify grapevine (*Vitis vinifera* L.) genotypes, while statistical evaluation of those has certain limitations, especially when we have no information about the traits that are discriminative to a certain sample set. High numbers of investigated characters could cause redundancy, while reducing those numbers may result in data loss. Grapevine is one of the most important horticultural crops, with many cultivars in production. The characterization of the genotypes is of undeniably high importance. In this study, we analyzed a dataset of scientific and historical importance with 125 morphological traits of 97 grapevine cultivars described by Németh in 1966. However, the traits are not independent in a set of a large number of categorical traits with too few cultivars. Therefore, the number of traits was first reduced using a simple and effective algorithm to eliminate traits with redundant information content using the asymmetric measure of association Goodman and Kruskal’s λ. We reduced the number of traits from 125 to 59 without any information loss. For the classification, we applied a random forest (RF) method. In this way, 93% of the cultivars were correctly classified using only four traits of the data set. To our knowledge, only a few studies applied a trait elimination algorithm similar to ours in ampelography that can be used for other biological data sets of similar structure. The classification results give a morphological explanation to several cultivars from the Carpathian Basin, a territory where all three *Vitis vinifera* L. geographical groups, occidentalis, orientalis and pontica, are represented. We found that the information-loss-avoiding data reduction method we applied in our study solved the redundancy-caused interdependencies and provided a suitable dataset for classifying grapevine genotypes. For example, this method may successfully be applied in digital image analysis-based traditional morphometric investigations in ampelography.

## 1. Introduction

Grapevine (*Vitis vinifera* L.) is one of the most widespread horticultural crops. The domestication of this liana-like plant dates back to 6000 B.C. in the Trans-Caucasus [1]. During the millennia of cultivation, the numbers of genotypes significantly increased, caused by natural mutation followed by selection and conscious cross-breeding. Today, there are approximately 10,000 registered cultivars in the world with various purposes of cultivation, such as wine, table grapes, raisin, and rootstock production [2]. From the beginning of the 19th century, botanical and viticulture literature aimed to classify a high number of genotypes according to morphological or phenological traits and geographical origin [3]. The main objectives of these studies were the identification of the cultivars to avoid synonyms that caused confusion in the wine sector and the appellation of the products. To classify grapevine genotypes, two main methods were applied during the past 200 years. The artificial classification is based on those morphological traits that could help the differentiation of the cultivars (berry color, berry shape, leaf lobature), while natural classification investigates the origin of the cultivars and those morphological and physiological traits which are possibly influenced by the place of origin for example bunch and berry size, trichomes (a.k.a. prostrate and erected hairs) and bud fruitfulness. All natural cultivars can be classified by this system since their phenotypic traits are caused by evolution and the ecological conditions they originate from.

Detailed taxonomic classification of the cultivated plant dates back to Linnaeus, de Candolle, and Alefeld. The later author introduced the term ‘variety group,’ which became *convariety* as a taxonomic expression. This category refers to the level between the subspecies and variety [4]. In the natural classification of the *Vitis vinifera* L. cultivars, this level has high importance. Negrul [5] classified cultivars according to eco-morphological traits into three *proles* (equivalent to the taxonomic level of the *convarietas*): *occidentalis, orientalis* and *pontica*, according to their putative origin based on the morphological similarities of the members within each of the groups [6]. According to this system, Németh [7,8,9] gave the most detailed classification of the Hungarian grapevine cultivars and applied a four-level taxonomic system: *convarietas, sub-convarietas, provarietas* and *sub-provarietas*. Convarietas *occidentalis* consists of Western-European cultivars such as Pinot noir, Chardonnay, Gamay, etc. Convarietas *orientalis* includes the Eastern (e.g., Middle-Asian) cultivars such as Chasselas, while the group *pontica* consists of cultivars with their origin of Georgia, Hungary, Greece, etc. such as Furmint, Gohér, and Ezerjó.

Certain morphological traits such as linear, angular parameters, and weight are described as continuous variables, while a number of lobes or seeds in the berry are introduced as ordinal variables. Part of the ampelographic characteristics is discrete/nominal variables such as the leaf or berry shape. A special type of variable is the dichotomic variable, which would have two values: most frequently, the presence or absence of an organ or feature. Numerical representation of morphological data is frequently applied in ampelography to provide international standards while blurring the intra-cultivar variability. Ravaz [10] already applied this methodology when continuous angular leaf morphometric data were transferred to classes providing categorical variables. Later Galet [11] and Németh [8,9] provided the ampelometric index for several cultivars, which was also based on categorical variables. Moreover, the descriptor list of the International Organization of Vine and Wine [12] and IPGRI, UPOV, and OIV [13] also code the different traits on ordinal, nominal, and dichotomous scales.

Discriminant analysis (DA) is a widely applied statistical method to investigate grape species, cultivars, or clones where continuous variables are the input. For example, Preiner et al. [14] showed that certain leaf phyllometric parameters provide high accuracy in correctly classifying grapevine cultivars. Later Bodor-Pesti et al. [15] reported high classification accuracy according to berry morphometric traits using digital image analysis. However, DA is developed only for continuous variables and does not work with categorical predictors. Instead, there are some multivariate classification methods applicable for categorical variables as well as for continuous ones (Classification And Regression Trees ‘CART’, Random Forest ‘RF,’ Multinomial Logistic Regression ‘MLR,’ Neural Networks ‘NN,’ Boosted Regression Trees ‘BRT’ and Gradient Boosting machines ‘GBM’) that have been successfully applied in cultivar characterization with biochemical [16], morphological [17,18,19] or NMR spectroscopy [20]. The random forest method (RF) is used for identification in biology [21], chemistry [22,23,24,25], morphometrics [26], and environmental studies [27].

With the spread of remote sensing in precision viticulture (PV), machine learning (ML) methods are frequently applied in big data management and evaluation processes in the classification of cultivars based on hyperspectral image analysis [28], in yield prediction [29] or molecular genetic investigations [30]. Aside from PV, morphological evaluation and identification of the genotypes can also be successfully performed by ML. Fuentes et al. [31] showed that ML is a powerful method in leaf morphological investigations. Later Landa et al. [32] showed the efficiency of ML in carpometric evaluations. Aside from the 2-dimensional evaluations based on elliptic Fourier analysis, recent 3D recordings and cross-section outline analysis of the samples proved efficient in discrimination [33,34].

In this study, 125 morphological traits of 97 cultivars reported by Németh [7] were analyzed to find an appropriate subset of morphological traits that explain the *convarietas* classification most successfully. Our objective was dual: (1) finding a trait selection method for data with a great number of dependent traits of ordinal and nominal types without information loss that can be step-wisely controlled by the user; (2) finding out how the selected traits can predict the three *convarietas* (*pontica, occidentalis*, and *orientalis*) into which the cultivars were classified by Németh, according to their geographical origin.

Previous research aiming at geographical origin and morphological trait-based classification is scarce in ampelography, while the information-loss-avoiding trait selection method can be used not only for this special data set but also in similar investigations.

We tested the hypothesis that (1) classification result is more easily interpretable by the original traits and (2) for this, redundancy reduction is possible to carry out without variable transformation and loss of information contained by the original data.

## 2. Results

### 2.1. Variable Selection of the Morphological Traits on the Examined 97 Cultivars

We evaluated the distribution of the categories of each trait. Among the 125 investigated traits, 52.8% (66 out of the 125) had three categories, and 21.6% had two categories. The traits with 4, 5, 6, and 7 categories were present in 11.2%, 9.6%, 0.8%, and 1.6%, respectively. The most diverse trait was the ‘density of the leaf blade’s hairs on the lower side’ with its nine categories. For example, the trait ‘young shoot’s (2–4 cm) color’ has five categories, but the trait ‘density of the young shoot’s (2–4 cm) hairs’ has only three. The numbers of categories are shown in section ‘Materials and Methods’, in Table 3 for all traits of the data set.

We found several relations between and among the morphological traits. Relations between or among certain determined trait—predictor trait(s) (i.e., one of them is completely determined by the other(s); λ = 1) are listed in Tables S1–S3. Note that the elicited traits are not necessarily less important than the others, but in this particular data set, they turned out to be replaceable by others. Reducing the redundancy of the data set is a mathematical necessity.

### 2.2. The Classification

As a second step to select the most important few traits out of the 59 remaining ones that can effectively discriminate between the three convarietas due to Németh, the RF method was carried out. The estimated proportion of the correctly classified cultivars reached 0.93 with three and four predictor traits and 0.94 with five predictor traits. Without variable selection, just 0.84 of the cultivars would be classified correctly. The best three-predictor-combination trait set is ‘density of the hairs on the lower side of the leaf,’ ‘width of the pith of cane internode,’ and ‘compactness of the unripe bunch,’ with 0.93 overall hit rate (0.96 in the *pontica*, 0.91 in the *occidentalis* and 0.86 in the *orientalis* group). Note that 0.86 represents the highest hit rate in the *orientalis* group with three predictor traits.

With four and with five predictor traits, the best solutions are represented in Table 1 and Table 2.

Notice that all four- and five-predictor-combination trait sets contain the best three-predictor combination trait set with different completions.

- The most often occurring traits are ‘density of the leaf blade’s hairs on the lower side,’ ‘width of cane’s internode’s pith,’ and ‘unripe bunch compactness.’
- The misclassified cultivars were the followings:
- cultivars of the pontica group, classified as *occidentalis*: Pozsonyi, Szagos kadarka;
- cultivars of the occidentalis group, classified as *pontica*: Malbec, Mourvèdre, Muscadelle;
- cultivars of the orientalis group, classified as *pontica*: Juhfark, Kékoportó.

## 3. Materials and Methods

### 3.1. The Data

In this study, we set out from a numerical phenotypic dataset of about 97 grapevine cultivars characterized by Németh [7] for 3 to 5 years. The ampelographic description was carried out according to 125 traits recorded on the cane (woody shoot), young shoot, shoot, young leaf, adult leaf, inflorescence, bunch, and berry (Table 3). Phenotypes of each trait were then numerically coded from 1 to 15 according to an ordinal or nominal scale. The original dataset contained cross-breed cultivars, omitted in our study to evaluate exclusively natural cultivars, classified into the convarietas: orientalis, occidentalis, and pontica with 7, 38, and 52 members, respectively. Lower levels of Nemeth’s classification system (*sub-convarietas, provarietas, sub-provarietas*) were not examined because of the low numbers of cultivars in the samples.

**Table 3:** The 125 morphological traits of *Vitis vinifera* L. cultivars, according to Németh [7] involved in our analysis, where the number of categories is given in brackets.

Organs	Traits with the Numbers of Their Categories
Shoot	
young shoot (2–4 cm)	color (5), density of the hairs (3)
young shoot (15–20 cm)	color (4), density of the hairs (4)
shoot tip	color (4), density of the hairs (4), goffering of blade (3)
tendril	color (3), density of the hairs (2), length (3), branching (2)
young cane	color of internode (3), color of node (3), density of the hairs of internode (5), density of the hairs of node (4), pattern of internode (5), waxiness of internode (3)
Leaf	
leaf petiole sinus	shape (3), depth (3), petiole sinus base limited by veins (2)
leaf upper lateral sinus	shape (3), width (3), shape of base (6)
leaf petiole	density of the hairs (9), color (3), pattern (3), cross-section (2), length (3), length compared to leaf blade (2)
leaf blade	width (3), shape (5), size (3), profile of blade in cross-section (4), goffering of blade (4), color (5), autumn coloration (6), depth of upper lateral sinuses (6), numbers of lobes (5), numbers of sinuses (5), shape of main lobe (3), shape of teeth (5), length of teeth compared with their width (5), density of the hairs of serrations (2), color of veins (4), angle between the veins (3), density of the hairs on the upper side (6), density of the hairs on the lower side (12), rigidity of structure (3), strength of structure (3), surface (2), gloss of surface (2)
other	angle between the leaf petiole and shoot (3), angle between the leaf petiole and blade (3)
trunk	vitality (3), density of shoots (3), attitude of shoots (3), shoot growing (2),
cane internode	density of the hairs (5), color (5), pattern (5), waxiness (3), structure of surface (3), length (3), width according to diameter (3), width according to perimeter (3), cross-section (2), width of pith (3)
cane node	color (2)
bud	position (3), density of the hairs (4), color (3), shape (3), size (3), covering of the bud (3)
Flower, inflorescence, bunch, berry and seed	
inflorescence	density of the hairs (2), color (3), branching (2)
flower	type (4), shape of pistil (5), size of pistil (3), angle of stamen (3), relative length of stamen (3)
unripe bunch	shape (5), compactness (3), density of the hairs on peduncle (2), color of peduncle (3), pattern of peduncle (2)
unripe berry	shape (4), color (5), pattern (3)
ripen bunch	shape (5), compactness (4), length (5), width (3), size (5), structure of peduncle (3), expansion of peduncle (3), length of peduncle (3), width of peduncle (3), pattern of pedicel (3), shape of pedicel (3), length of pedicel (3), width of pedicel (3)
ripen berry	ease of detachment from pedicel (2), shape (15), cross-section (3), length (5), width (5), size (5), color (8), pattern (5), waxiness (2), pulp (4), color of pulp (3), taste (4), color of brush (3), length of brush (3), thickness of skin (3), structure of skin (3), shape of seed (4), size of seed (3), color of seed (5), shape of the seed body (3), length of the seed beak (3)
other	ripening time (5)

### 3.2. Interdependencies between the Morphological Traits

The 125 morphological traits showed many interdependencies, typical in data analysis with many traits and relatively few cases. Data with related variables contain redundant information.

Therefore, we started reducing the rate of redundancy contained by the traits. Dimension reduction (DR) methods like principal component analysis (PCA) or discriminant analysis (DA) are often successfully used for dimension (and interdependency) reduction preceding genotype classification according to morphology, moreover, morphometric traits [35], however, they are developed for continuous traits, and it is not easy to interpret the importance of the individual traits of the final model.

Variable selection (VS) methods keep a subset of the original traits, and they are based on association or mutual information rate [36]. Note that DR and VS methods result in some information loss, though at a low level. Moreover, since VS is controlled by a measure (e.g., association), the user is usually not involved in omitting a trait.

In what follows, we describe a variable selection method

(1) that preserves all information contained by the original data set while traits are omitted if one or more other traits completely predict them;
(2) that can be step-wisely controlled by the users and
(3) which can also manage asymmetric association (a trait X predicts Y, but Y cannot predict X).

We found two traits—tendril’s branching and leaf petiole’s cross section—which were constant in the whole data set, so they could not have been applied for discrimination between the groups; we omitted them. Having constant traits was not surprising since ampelographic descriptions should be suitable for all *Vitis* species and cultivars. Thus, in the case of *Vitis vinifera* L., e.g., the tendril’s branching is constant with two branches, in contrast to some other *Vitis* species with tertiary branches.

There were a lot of weaker or stronger dependencies between the 123 traits, suggesting that the number of traits could be reduced without any information loss.

For computational feasibility, we chose the strategy to omit step-by-step those traits which were completely predictable by some others. First, those were predictable by a single trait, then those were predictable by two, and so on, up to four predictors.

To ascertain complete predictability, we used Goodman-Kruskal’s λ [37], which is a measure of proportional error reduction for categorical variables (in our case: traits). Lambda (λ) is defined by the formula $\lambda \left(Y|X\right)=\frac{\varepsilon_{1}-\varepsilon_{2}}{\varepsilon_{1}}$, where $\varepsilon_{1}$ and $\varepsilon_{2}$ are the error rates of prediction of the dependent variable Y if we know ($\varepsilon_{2}$) and if we do not know ($\varepsilon_{1}$) the value of the independent variable X. Lambda (λ) measures the association on a scale from 0 to 1; λ(Y|X)=1 means that Y is completely determined by X (i.e., $\varepsilon_{2}=0$, so we can predict Y exactly if we know X) while λ(Y|X)=0 means that Y is not better predictable even if we know X (i.e., $\varepsilon_{1}=\varepsilon_{2}$, so we can predict Y with the same error rate if we know or do not know X). Note that lambda is not symmetric; that is, λ(Y|X) may differ from λ(X|Y). For independent variables X and Y, λ(Y|X)=λ(X|Y)=0, but the converse does not hold: if λ(Y|X)=0, the variables Y and X are not necessarily independent. It can happen that Y is not predictable from X but X is predictable from Y (we will show such an example later).

First, we detected the situations when one trait determined another (λ = 1, Table 4). We found flower type, the shape of the unripe bunch, and the adhesive ability to the pedicel of berry as the traits which could be omitted because either filaments of the flower or the length of the flower’s filaments determine flower type, while the diameter of cane determines the adhesive ability to the pedicel of the berry, and shape of bunch and shape of unripe bunch determine each other. We chose the ‘shape of bunch’ to keep it in the data set. As each step of the variable selection can fully be controlled by experts, if the aim of the study is especially the identification of the variety at an earlier stage during the vegetation, the shape of an unripe bunch to keep can be another reasonable choice.

Since Goodman-Kruskal’s λ can handle two variables only, in the case of learning the relationship of more than two traits, it was necessary to create unified new variables from two or more original ones such that each level combination of the categorical values of the traits is represented by a different value of the new one. A new variable $V_{new}$ is created from two predictor traits by combining their category levels e.g. if trait $T_{1}$ has three category levels (1, 2, 3) and trait $T_{2}$ has two category levels (1, 2): $V_{new}=V_{T_{1}T_{2}}$ has 2 × 3 = 6 possible category levels, as shown in Table 5. Note that from the possible 6 category levels, we defined only those that really occurred in the dataset. Then, to examine the predictability of trait T by the traits $T_{1}$ and $T_{2}$, the Goodman-Kruskal’s λ were calculated as $\lambda \left(T|V_{T_{1}T_{2}}\right)$.

If a trait was completely determined by others, it was eliminated from the data set. By doing this, we reduced the number of traits step by step. We found several trait pairs, the combination of which perfectly determined another trait of the data set. Since we cannot give the whole list because of their rather large number, we give an example in Table 6:

In Table 7, we show an example of how the cane’s density of hairs can be determined by the density of hairs of the shoot tip and the density of hairs of the young shoot. (Note that the predicting traits in this example are very important ones discussed later).

Inspecting all the twin cases (a trait determined by two other ones), we realized that we could eliminate a determined trait only if we do not need that one for predicting another trait. Finally, we could leave out the following five traits (determined by two other ones) without any information loss (Table S1).

We continued this process and omitted 19 further traits that were perfectly determined by a triplet of the remaining traits (Table S2).

In the next round, we managed to leave out the following 36 traits as they were perfectly determined by four other traits (Table S3).

The process can be continued until the number of traits is sufficiently low for an effective application of a classification method. We stopped the reduction process after having eliminated 66 traits.

Note that although some of the relations we found might appear just in this particular dataset, the reduction was necessary to make the follow-up procedures (e.g., discrimination power exploration) more effective and reliable. However, in the case of another data set, the algorithm we used to eliminate the traits with redundant information can be applied the same way.

With the remaining 59 traits, we performed a random forest method.

### 3.3. The Classification Method

For the classification, we applied the random forest (RF) method, using the packages rpart [38], nnet [39], randomForest [40,41], and caret [42] of the statistical software R 4.2.1 [43].

Random forest is a generalization of CART (Classification And Regression Trees [44]). CART is a recursive method; in each step, it finds the variable (in our case: trait) which splits the set of cases best fitting to the original classification. In this way, it creates branches, and either branch can be split again in the next step. This method is very effective but has the weakness that it is hierarchical: if a variable seems important in the first step, it will remain in the classification even if some other variables could easily take over its role. That is why we prefer the random forest method.

The random forest method is a machine learning algorithm for classification and regression [45], i.e., for modeling a classification by predictor variables. As a supervised machine learning algorithm, it uses a training subset of the original dataset (in our case: a subset of cultivars) for a preliminary classification to learn and formulates a model explaining the classification by the predictor variables (traits). As a next step, after the “training” process, it considers the remaining subset (test subset) of the original dataset (in our case: a subset of cultivars) to test the model. In contrast to CART producing only tree output, RF builds hundreds of trees (set to ntree = 500) and summarizes their results to make the consensus prediction stronger than simple CART models. The RF consensus decision is based on the classification confirmed by the majority of the trees.

The RF trees are random since, in each step of the algorithm, a random sample from the whole training dataset is taken with replacement. The dataset that is not used in a step (approximately 1/3) is called out-of-bag (OOB). A great advantage of RF is that it is not sensitive to outliers and missing data [46]. Summarizing the predictions of a great number of random tree outcomes, with an appropriately tuned length of the trees, RF can avoid or at least considerably reduce overfitting, the typical weakness of other tree-based methods. Overfitting occurs when the model mirrors the analyzed particular set of data rather than the true nature of the modeled structure, and therefore, the developed model is not appropriate to explain another data set; therefore, it is unsuitable for reliable further conclusions or generalizations. The accuracy of the RF method was evaluated by cross-validation [47]. The model fitted by a randomly selected part of the cultivars (training set) was evaluated on the rest of the cultivars (test set). Accuracy of the classification was defined as the rate of correct classifications in the test set (the so-called hit rate, i.e., the ratio of the cases when the model prediction of group membership is the same as the original group membership). By repeating this procedure, a sufficient number of times by randomly splitting the data set into the training set and test set, a good estimate of the accuracy of classification can be obtained.

In our case, since the orientalis group has only seven cultivars (out of the 97 ones), the simple random partitioning into training and test sets would be inappropriate because it might often happen that the orientalis group would lie even fully within one part (fully within the training, or fully within the test set). For example, for a split of a training set of 80 cultivars with a test set of 17 ones, the probability of having all orientalis cultivars into the same set is 25%, and for a split of 70 + 27 cultivars, it is still as high as 9%. If the orientalis group is fully in the test set, with no cultivar in the training set, the fitted model will be unable to explain this group correctly. On the other hand, if it is fully in the training set, then the result of cross-validation reflects solely on the classification of the other two groups. Therefore, to provide that each group is represented proportionally in both the training and test set, we decided to make the random partitioning separately by stratified sampling [48]. We worked with test sets of size 13, with seven cultivars of the pontica, five occidentalis, and one orientalis class. So, the smallest group could be represented in the training set with six cultivars.

A cross-validation run was carried out with 2000 replications. This means that the hit rates are estimated from 14,000, 10,000, and 2000 output values for the pontica, occidentalis, and orientalis groups, respectively, resulting in acceptable precision (the Standard Error of the estimate is below 1% for the smallest group and is below 0.5% for both the larger groups).

Conjecturing that the classification power (i.e., the hit rate) may already be fairly acceptable with merely three predictor traits, all combinations of three traits were tried. After that, the best three trait combinations (those with a hit rate greater than 0.8) were completed with each trait set to get four predictor trait models. Finally, the best four- and five-trait combinations of models were selected and are reported here.

## 4. Discussion

The origin of the grapevine and classification of the large numbers of cultivars are the focus of many studies. According to Andrasovszky [49], *Vitis vinifera* L. is not a single species but cultivars belonging to five different species. Hegi [50] assumes that *Vitis vinifera* L. has two subspecies as subsp. *sylvestris* (Gmel.) and subsp. *sativa* (DC.). The classification of grapevine cultivars according to their geographic origin dates back to Odart [51], Andrasovszky [49], Marton [52], and Negrul [5]. The latter author defined three *proles*: *orientalis*, *occidentalis*, and *pontica* and classified the cultivars into these. This later theory was continued by Németh [8], who described the large numbers of Hungarian cultivars based on numerical systematics and classified them into three *convarietas*.

Finding discriminative morphological traits and classifying cultivars according to their geographical origin has not only agro-historical importance but, according to Negrul, it would improve the success of breeding ([53] and citations therein). Moreover, learning that the origin of the genotypes could influence the resistance against certain pathogens [54] would serve the breeding of new cultivars and provide a lower environmental impact on the cultivation.

Several studies investigated the link between morphological or agro-biological traits. Averna-Saccá (cit. in: [55]) found a positive correlation between the yield, sugar content, acidity of the must, and the angles between the veins of the leaf. Bodor-Pesti et al. [15] also found a positive and significant correlation between seed number and berry size traits.

Correlation among the traits has high importance in molecular linkage maps and QTL analysis. A former study showed that certain phenological and morphological traits significantly correlate positively and negatively. Mapping of the population of F1 progenies obtained from the cross between Italia and Big Perlon revealed a positive correlation between veraison length and mean seed number and between mean berry weight and seed weight. While the correlation between mean seed number and dry matter was negative [56]. Note that the link between certain traits is simply caused by the fact that those are investigated on the same organ but at different phenological stages, and the trait is not changing between the two observations. For example, in our study, the shape of the unripe and ripe bunches was included. They refer to the same trait measured at different time points and are obviously interrelated. The shape of the bunch (architecture and length of internodes in the rachis) did not change between the two inspection dates; only its size increased while phenotyping was carried out by Németh [7].

As demonstrated in several studies, traits are usually interrelated, meaning they are not independent. It can cause redundancy and difficulties in statistical analysis [57]. The other problem is that large numbers of traits are usually causing difficulties in investigating and interpret. For this reason, for example, OIV [12] also reduced the numbers of the traits in the grapevine descriptor list and highlighted the most important ones that can serve as identification of the cultivars. Redundancy in the data set makes the statistical analysis very cumbersome. Predictor traits with nearly the same information content are interchangeable, resulting in lots of equivalent solutions. Dimension reduction or variable selection can help to increase the stability of the models. Our variable selection process resulted in lower misclassification rates between the pontica and occidentalis groups. By the random forest method, subsets of traits were found that classified the cultivars with a success rate of 0.94. Even the most misclassified group (orientalis) was separable. A success rate of 0.86 in this group means that, on average, one of the seven cultivars is misclassified. With four predictor traits, the overall accuracy was the same, but it resulted in a slightly higher hit rate on the orientalis group. These results partly overlap with Németh [8], who stated that the trichomes on the shoot tip and the leaf are the main traits defining the membership in each convarietas. Our statistical evaluation did not verify the importance of the shoot tip, while further traits of the cane and unripe bunch are valuable in the classification.

Comparisons of the natural classification of different authors sometimes show contradictions. Lőrincz et al. [58] introduced the classification provided by Marton and Negrul and highlighted the main differences between them. Namely, Marton misclassified several cultivars, for example, Juhfark, which were misclassified in our study too. These types of misclassifications could be explained by the fact that cultivars were investigated in different geographical regions by different authors, and the effect of the terroir would have a significant effect on the morphological traits as it was examined, for example, in Somogyi et al. [59]. Vineyard maintenance also has a noticeable effect on the phenotype, as shown by Intrieri et al. [60]. These reasons could explain why some authors classified cultivars into different groups based on morphological traits.

Recent morphometric evaluations according to elliptic Fourier description outline analysis and landmark-based generalized Procrustes analysis of large numbers of grapevine and wild grape (*Vitis sylvestris* C.C. Gmel Hegi.) leaf samples showed high intra- and interspecific diversity. In their study, Chitwood et al. [61] grouped the investigated grapevine cultivars with “western,” “central,” and “eastern” indications and found a significant correlation between certain traits with the origin of the accessions, like complex of hirsuteness, angular traits of the veins, lobing, and serrations. These results underline our findings, as trichomes of the accessions are important traits in investigating the geographical origin of grapevine genotypes.

The variable selection method we applied can be useful in general in biological classification. In the case of grapevine, the study of other datasets would be promising, especially those where genetic and morphological data are both available. The introduced result of this special dataset is particular; however, the dataset is rich in cultivars from the Carpathian Basin, especially from Hungary, a territory where all the three *Vitis vinifera* L. geographical groups, —Negrul’s *proles* or *Vitis vinifera* L. *convarietas*—*occidentalis*, *orientalis* and *pontica* are represented. Within these geographical groups, further classifications are possible, as done by Negrul [5] and Németh [7]; however, this approach was not targeted in the present study.

## 5. Conclusions

Our study showed that variable selection from categorical ampelographic data based on Goodman-Kruskal’s λ is a powerful method to avoid high-level interdependencies with no information loss. Classification of grapevine genotypes according to the geographic origin based on the reduced dataset resulted in high accuracy. These results underline the importance of certain morphological traits that show discriminative power in natural classification. Our results can be inspiring not only for researchers in ampelography but also for others working on category-type-data-based classification.

## Figures and Tables

**Table 1:** The rate of correct classifications in the test set (overall hit rate) and the subsets pontica, occidentalis, and orientalis resulted in using four predictors with the random forest method. The best three-predictor-combination set is in italic.

Predictor Traits	Hit Rate			
	Overall	*Pontica*	*Occidentalis*	*Orientalis*
*density of the hairs on the lower side of the leaf* shoot density of the trunk *width of pith of cane internode* *compactness of the unripe bunch*	0.93	0.97	0.89	0.87
*density of the hairs on the lower side of the leaf* *width of pith of cane’s internode* *compactness of the unripe bunch* ripening time		0.96	0.89	0.87
goffering of the leaf blade *density of the hairs on the lower side of the leaf* *width of pith of cane internode* *compactness of the unripe bunch*		0.95	0.91	0.87

**Table 2:** The rate of correct classifications in the test set (overall hit rate) and the subtests pontica, occidentalis, and orientalis resulted in using five predictors with the random forest method. The best three-predictor-combination set is in italic.

Predictor Traits	Hit Rate			
	Overall	*Pontica*	*Occidentalis*	*Orientalis*
*density of the hairs on the lower side of the leaf* vitality of the trunk *width of pith of cane internode* *compactness of the unripe bunch* length of the ripen bunch	0.94	0.96	0.92	0.83
*density of the hairs on the lower side of the leaf* pattern of cane’s internode *width of pith of cane’s internode* *compactness of the unripe bunch* pattern of the pedicel of ripen bunch	0.93	0.96	0.90	0.87

**Table 4:** Traits that are fully determined by another trait together with the direction of determination.

Predictor Traits		Determined Traits
cane’s internode’s width according to diameter	→	ease of detachment from ripen berry’s pedicel
angle of flower’s stamen	→	flower’s type
relative length of flower’s stamen	→	flower’s type
ripen bunch’s shape	↔	unripe bunch’s shape

**Table 5:** An example how the values of a new variable $V_{new}=V_{T_{1}T_{2}}$ can be created by combining the categories of traits $T_{1}$ with three and trait $T_{2}$ with 2 levels.

Traits	Values of the Traits					
$T_{1}$	1	1	2	2	3	3
$T_{2}$	1	2	1	2	1	2
$V_{T_{1}T_{2}}$	1	2	3	4	5	6

**Table 6:** Two examples when two traits determine another trait perfectly.

Predictor Trait 1	Predictor Trait 2		Determined Trait
density of hairs of shoot tip	density of hairs of young shoot	→	canes density of hairs
taste of berry	density of hairs of young shoot	→	canes density of hairs
		→	canes density of hairs

**Table 7:** An example of how two traits can determine another trait perfectly.

	Predictor Trait 1		Predictor Trait 2		Determined Trait
If	density of the young shoot’s (2–4 cm) hairs	and	density of the shoot tip’s hairs	then	density of the inflorescence’s hairs
	1		1 or 2		1
	1		3 or 4		no cases
	2		1		no cases
	2		2 or 3 or 4		1
	3		1		no cases
	3		2		1
	3		3 or 4		2

## Supplementary Materials

The following supporting information can be downloaded at: https://www.mdpi.com/article/10.3390/plants11243428/s1, Table S1: Traits that were eliminated because they were perfectly determined by two other traits. Table S2: Traits that were eliminated because they were perfectly determined by three other traits. Table S3: Traits that were eliminated because they were perfectly determined by four other traits.
