# Supplementary material for: Morphological Traits Evaluated with Random Forest Method Explains Natural Classification of Grapevine (Vitis vinifera L.) Cultivars
Source: Plants (Basel). 2022 Dec 8;11(24):3428. doi: 10.3390/plants11243428 (PMC9781146; doi:10.3390/plants11243428)
Supplement: Supplementary file 1 [file plants-11-03428-s001.zip › plants-2031411-supplementary.pdf]

Table S1. Traits that were eliminated because they were perfectly determined by two other traits.

leaf petiole's length compared to leaf blade	density of the inflorescence's hairs
numbers of leaf blade's sinuses	expansion of ripe bunch's peduncle
density of canes internode's hairs	

Table S2. Traits that were eliminated because they were perfectly determined by three other traits.

young shoot: (15-20 cm) density of hairs	unripe berry: shape
tendril: color	bunch : width
bud of young shoot: density of hairs	pedicel: width
mature leaf: petiole sinus base limited by veins	berry: cross section
mature leaf: shape of the tip of main lobe if	berry: size
mature leaf: density of hairs on the upper side of blade	berry: patterning
mature leaf : sanity	berry: flesh
nodes: color	berry flesh: color
bud: color	seed: shape
peduncle of unripe bunch: color	

Table S3. Traits that were eliminated because they were perfectly determined by four other traits.

young shoot (2-4 cm): density of hairs	cane: bloom
young shoot (2-4 cm): color	cane: diameter
tendril: density of hairs	cane: cross section
young shoot: color	bud: size
young shoot: color of the bud	florescence: color
mature leaf: shape of upper sinus	ovary flower: size
mature leaf: shape of base of upper sinus	unripe bunch: density of the hairs on peduncle
mature leaf: color of petiole	unripe bunch: patterning of peduncle
mature leaf: pattern of petiole	unripe berry: patterning density of bunch
mature leaf: length of petiole	bunch: width of peduncle
mature leaf: width	berry: shape
mature leaf: size	berry: bloom
mature leaf: number of lobes	berry: taste
mature leaf: density of hairs on teeth	berry: brush color
mature leaf: angles between the veins	berry: brush length
mature leaf: strength of lamina	seed: berry size
shoots curly	berry: shape of seed
angle between shoot and petiole	berry: length of stalk
